# The reliability and validity of gait analysis system using 3D markerless pose estimation algorithms

**DOI:** 10.3389/fbioe.2022.857975

**Published:** 2022-08-10

**Authors:** Shengyun Liang, Yu Zhang, Yanan Diao, Guanglin Li, Guoru Zhao

**Affiliations:** ^1^ CAS Key Laboratory of Human-Machine Intelligence-Synergy Systems, Research Center for Neural Engineering, Shenzhen Institute of Advanced Technology, Chinese Academy of Sciences, Shenzhen, China; ^2^ Shenzhen College of Advanced Technology, University of Chinese Academy of Sciences, Shenzhen, China; ^3^ Guangdong-Hong Kong-Macao Joint Laboratory of Human-Machine Intelligence-Synergy Systems, Shenzhen Institute of Advanced Technology, Chinese Academy of Sciences, Shenzhen, China

**Keywords:** single-camera video, 3D markerless pose estimates, 3D marker-based motion analysis, validity, reliability

## Abstract

Quantifying kinematic gait for elderly people is a key factor for consideration in evaluating their overall health. However, gait analysis is often performed in the laboratory using optical sensors combined with reflective markers, which may delay the detection of health problems. This study aims to develop a 3D markerless pose estimation system using OpenPose and 3DPoseNet algorithms. Moreover, 30 participants performed a walking task. Sample entropy was adopted to study dynamic signal irregularity degree for gait parameters. Paired-sample t-test and intra-class correlation coefficients were used to assess validity and reliability. Furthermore, the agreement between the data obtained by markerless and marker-based measurements was assessed by Bland–Altman analysis. ICC (C, 1) indicated the test–retest reliability within systems was in almost complete agreement. There were no significant differences between the sample entropy of knee angle and joint angles of the sagittal plane by the comparisons of joint angle results extracted from different systems (*p* > 0.05). ICC (A, 1) indicated the validity was substantial. This is supported by the Bland–Altman plot of the joint angles at maximum flexion. Optical motion capture and single-camera sensors were collected simultaneously, making it feasible to capture stride-to-stride variability. In addition, the sample entropy of angles was close to the ground_truth in the sagittal plane, indicating that our video analysis could be used as a quantitative assessment of gait, making outdoor applications feasible.

## Introduction

Gait parameters have been proposed as an index of overall gait pathology for elderly people. It uses kinematic and kinetic variables to be taken as a cleaner reflection of gait quality (Brach et al., 2001). Kinematic analysis is often performed in laboratory research using three-dimensional motion capture or wearable sensors, which are expensive, immobile, data-limited, and require expertise (Cronin, 2021). Recently, video-based pose estimation suggests the potential for analyzing gait kinematic parameters (Andriluka et al., 2014).

Video-based 2D body pose estimation is a well-studied problem in computer vision, with state-of-the-art methods being based on deep networks (Toshev and Szegedy, 2014; Fang et al., 2017; Güler et al., 2018). With the advent of deep neural networks, it is now possible to estimate joint angles without the need for reflective markers. One of the more popular approaches, CMU’s OpenPose enables key body landmarks to be tracked from multiple humans in a video in real-time (Cao et al., 2017). Yagi et al. (2020) used OpenPose to detect multiple individuals and their joints in images to estimate step positions, stride length, step width, walking speed, and cadence, in comparison with multiple infrared camera motion capture system OptiTrack (Lénárt et al., 2018). Kidziński et al. (2020) designed machine learning models (e.g., convolutional neural networks, random forest, and ridge regression models) to predict clinical gait metrics based on trajectories of 2D body poses extracted from videos using OpenPose. Similarly, Stenum et al. (2021) used OpenPose to compare spatiotemporal and sagittal kinematic gait parameters of healthy adults against recorded optical marker–based motion captured from walking simultaneously. These previous studies have been performed in comparison between markerless and marker-based methods; however, they only learned to infer joint angles or joint locations in the sagittal plane.

Some researchers have detected 3D skeletons by existing 2D human pose detectors from images/video (Martinez et al., 2017; Moreno-Noguer, 2017; Nie et al., 2017) by directly using image features (Zhou et al., 2016; Zhou et al., 2018). Martinez et al. (2017) used a relatively simple deep feed-forward network to lift 2D pose to 3D pose efficiently based on given high-quality 2D joint information. Nakano et al. (2020) compared joint positions estimated from the 3D markerless motion capture technique based on OpenPose with multiple synchronized cameras against recorded three-dimensional motion capture. Multicamera systems are not easy to deploy in real-life environments. Instead, we try to adopt the OpenPose to computer the 2D pose input to our 3DPoseNet based on a single-camera system and then estimate gait parameters in sagittal, coronal, and transverse planes.

The main contributions of this work include 1) novelty: unlike previous studies, we did not directly extract the trajectories of 2D body poses to predict gait metrics using machine learning models. Instead, we try to estimate the 3D human pose from video to measure gait parameters. Then, we clarify associations and agreements of motion analysis using markerless and marker-based systems and confirm the reliability and validity of 3D human pose from video. 2) Dataset: we have collected the synchronized motion capture cameras and a single-camera video dataset of movement sequences for elderly and young people. 3) Application: our video-based gait analysis workflow is freely available, involves minimal user input, and does not require prior gait analysis expertise.

## Methods

In the laboratory workflow, participants were marked with a total of 39 markers placed on bony landmarks. The Body39 joints were labeled by the Plug-In Gait full body model in Nexus software. Then, participants were required to perform the clear step on the treadmill, keeping 3 s and repeating three times. The positions of these markers were tracked by several optical cameras, which were later reconstructed into 3D position time series. Measures derived from the Vicon data served as ground_truth labels. In our proposed workflow, we used a camera to record the participant’s movement. The open-source OpenPose algorithm was adopted to extract trajectories of 2D key points from continuous images. The 2D joint positions from OpenPose were used as input, and we adopted a 3D keypoint detection algorithm (3DPoseNet) to estimate body joint locations in 3-dimensional space. It was important to note that these two workflows were synchronized by hardware. Finally, these signals from two workflows were converted to joint angles as a function of time.

### Experimental setting

**FIGURE 1:**
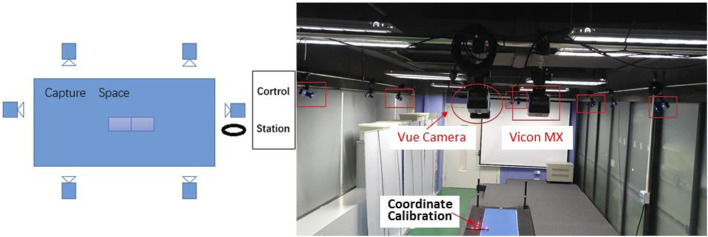
Overview of the experimental environment and setup.

Figure 1 showed the laboratory environment and setup, allowing us to capture data from seven sensors (six Vicon MX motion capture cameras and one Vue video camera). The designated laboratory area was about 5 m × 8 m × 3 m, where participants were fully visible in all cameras. The motion capture cameras were rigged on the wall shelf, four on each left and right edge and two roughly mid-way on the horizontal edges, for recoding three-dimensional marker trajectories at 60 Hz (Liang et al., 2021). The video camera was also rigged on the wall shelf, for recording images of the walking sequences at 60 Hz. The camera images were RGB files with a 1920 × 1080 pixel resolution. Multiple infrared cameras and digital video camera recording were used for hardware synchronization so that each time point of the motion capture data point corresponded to the time of every video frame.

**FIGURE 2:**
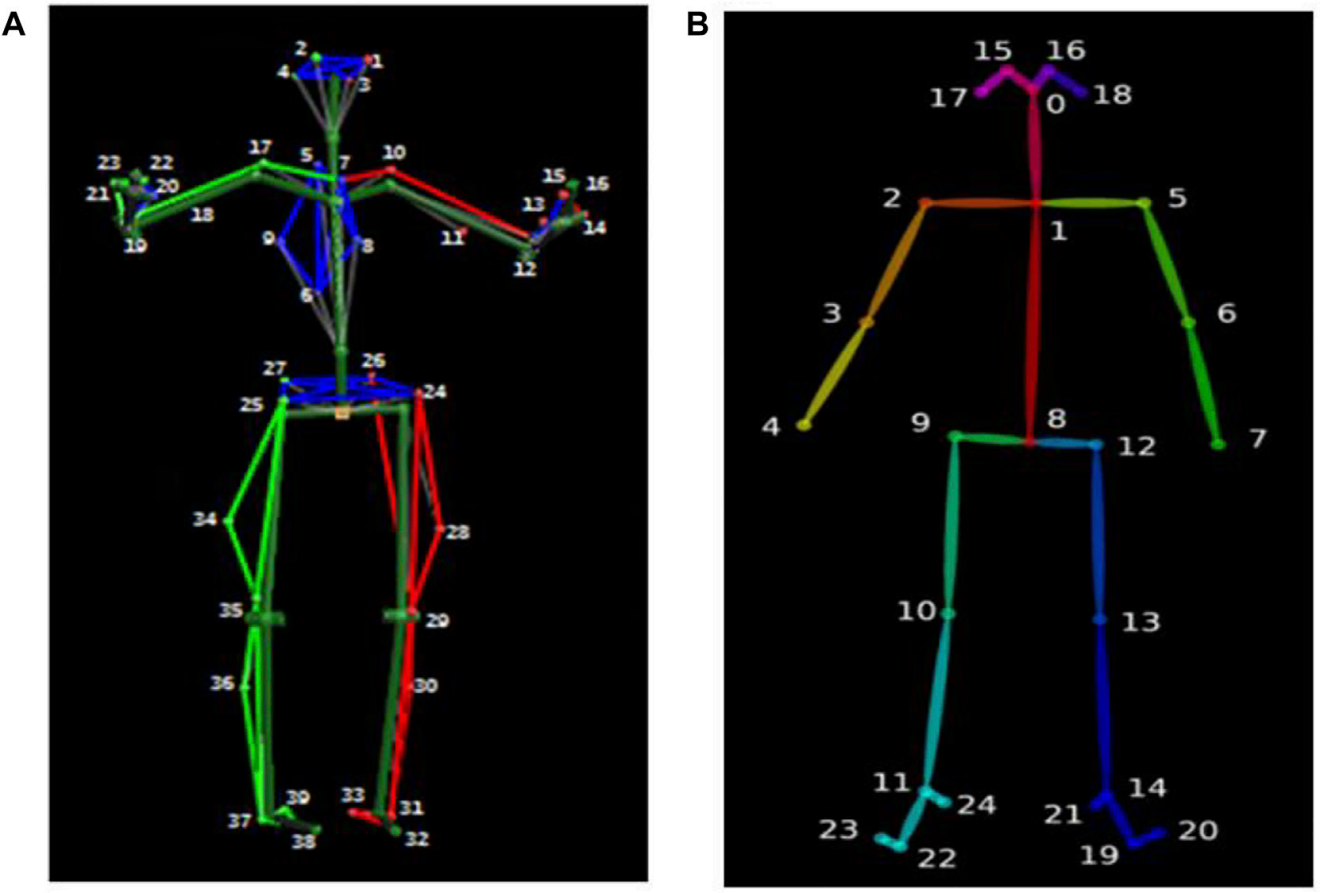
Body39 joints are based on the Plug-In Gait full body model **(A)** and OpenPose Body25 keypoint model **(B)**.

Moreover, 15 healthy elders [mean (SD) age: 56.6 (2.53) years] and 15 healthy young people [mean (SD) age: 27.27 (4.31) years] wore minimal, close-fitting clothes and participated in walking on the treadmill. The speed was set at 1.5 km/h for the younger and 0.8 km/h for the elderly. Motion capture was recorded by tracking 39 markers, Figure 2A showed the Body39 joints labeled by the Plug-In Gait full body model in Nexus software (Vicon®, 2002). The standard protocols of setting markers were designed to match the skeletal configuration of the Human3.6M dataset (Ionescu et al., 2014).

### Extracting 2D keypoints with OpenPose

The OpenPose algorithm first estimates features from each image using a 10-layer VGG19 network (Cao et al., 2017). In addition, the obtained feature map is put into two convolutional neural networks for calculating the confidence and affinity vectors for each key point. The heat maps with confidence and with affinity fields are obtained. Then, the 2D body joint locations are clustered, according to dichotomy matching in graph theory. The nonparametric representations called part affinity fields are used to regress joint position and body segment connections between the joints. Finally, the output has the confidence of prediction and X and Y pixel coordinates. Figure 2B shows the Body25 joints labeled by the OpenPose body model.

### Extracting 3D keypoints with 3DPoseNet

After obtaining 2D detections using OpenPose, our goal is to estimate 3D body joint locations. Formally, our input is a series of 2D points $x\epsilon {\mathbb{R}}^{2n}$ , and our output is a series of points in 3D space $\;y\epsilon {\mathbb{R}}^{3n}$ . We aim to learn a function $\;f^{*}:{\mathbb{R}}^{2n}\to {\mathbb{R}}^{3n}$ that minimizes the prediction error over a dataset of *N* poses:

$$f^{*}=\underset{f}{\min}\frac{1}{N}\sum_{i=1}^{N}\ell \left(f\left(x_{i}\right)-y_{i}\right),$$ (1)

where $x_{i}$ is 2D joint obtained from the output of OpenPose. $f^{*}$ is a deep and multilayer neural network with batch normalization (Ioffe and Christian, 2015), dropout (Srivastava et al., 2014), rectified linear units (RELUs) (Nair and Hinton, 2010), and residual connections (He et al., 2016).

**FIGURE 3:**
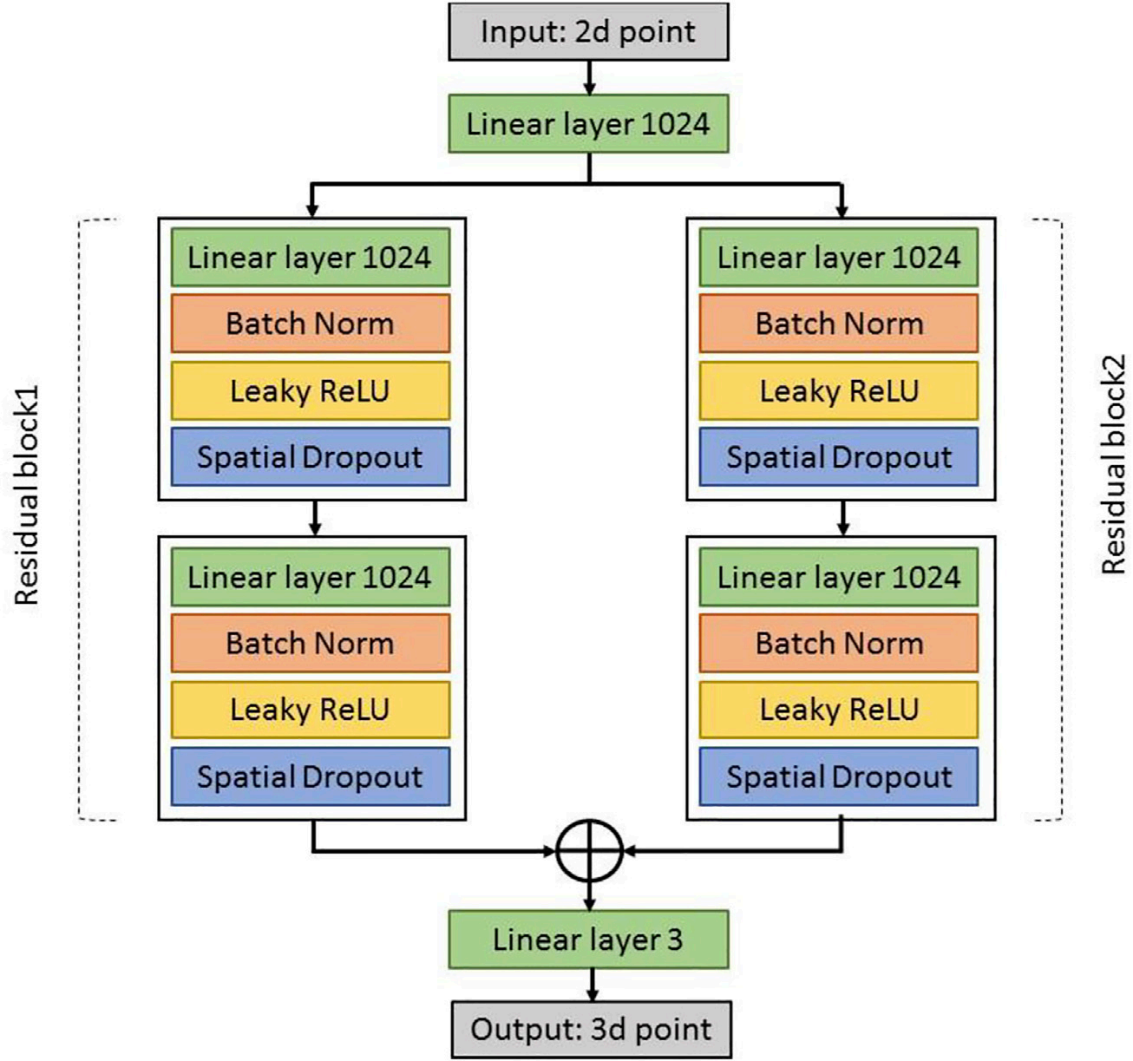
Diagram with the basic building blocks of 3DPoseNet.

Figure 3 shows a diagram with the basic building blocks of 3DPoseNet. The network is a multilayer convolutional neural network which inputs an array of 2D joint positions and outputs a series of joint positions in 3D. First, the linear layer applies directly to the input, which increases its dimensionality to 1024. Then, there are two same residual blocks. Each block includes two linear layers, Batch Norm, RELUs, and Dropout, with residual connections. Before the final prediction, the linear layer can be applied to produce outputs of size 3n. Initially, the weights of our linear layers are set using Kaiming initialization (He et al., 2015). The 3DPoseNet is trained for 200 epochs using Adam (Kingma and Ba, 2014), a starting learning rate of 0.001 and exponential decay, using mini-batches of size 64. In addition, we first train the network using the Human3.6M dataset (Ionescu et al., 2014).

### Gait parameter extraction

**FIGURE 4:**
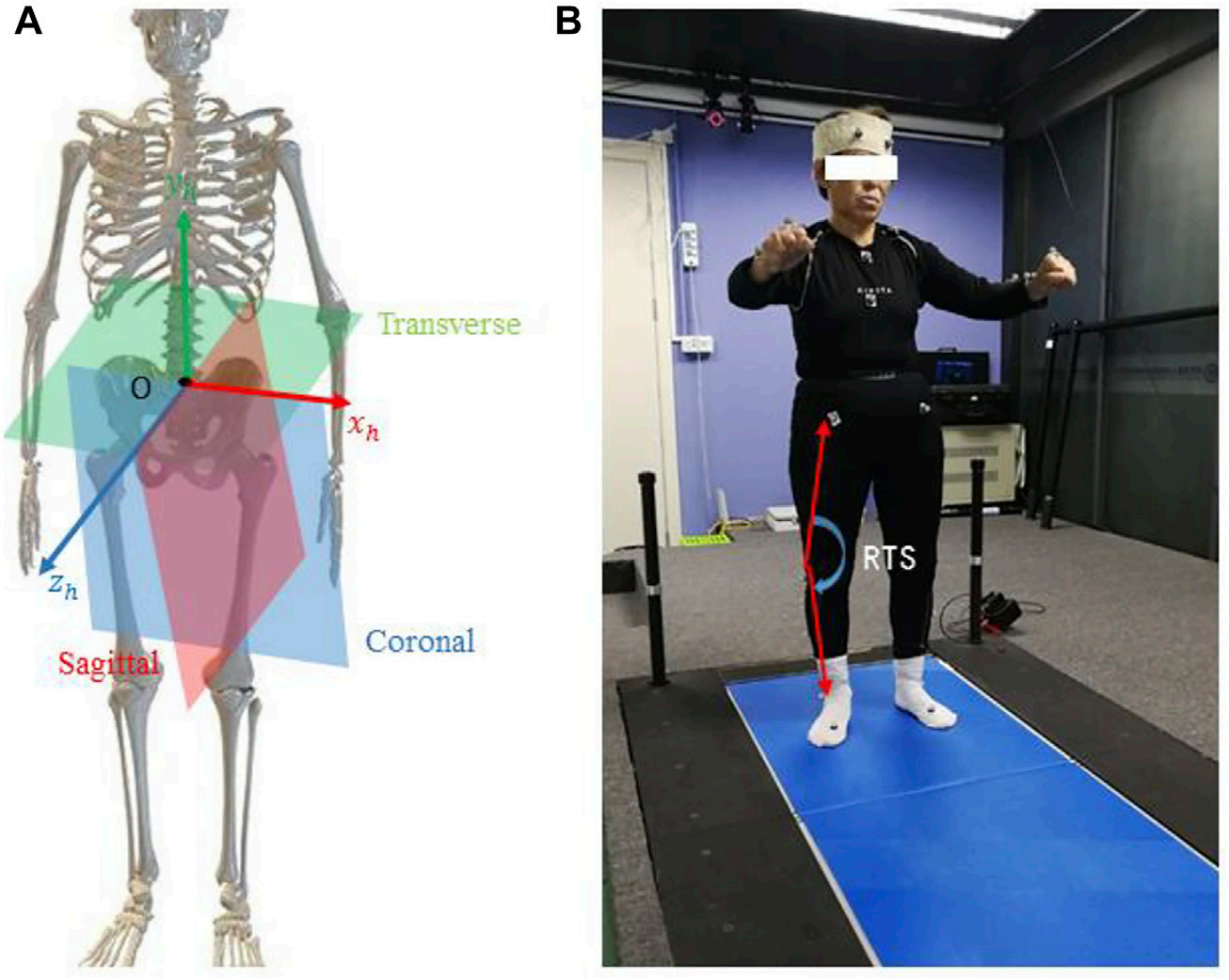
Illustration of the human body coordinate system **(A)** and extracted joint angle features for the subject **(B)**.

First of all, we filled gaps in keypoint trajectories using linear interpolation and smoothed trajectories using a one-dimensional unit-variance Gaussian filter. It should be noted that the markerless-based system returns 3D coordinates resolved in a local system around the middle of the hip joint. The 3D coordinates provided by the marker-based system came from a 3D global coordinate reference system fixed on the ground. These two different coordinate systems were moved to a new, coincident human-based local coordinate system {O}, as shown in Figure 4A. We centered each univariate time series by subtracting the coordinates of the pelvis and scaling all values by dividing them by the Euclidean distance between the right hip and the right shoulder.

The definition of the coordinate system was as follows: X was anterior/posterior, Y was lateral/medial, and Z was inferior/superior. The alignment procedure was taken from the study by Kabsch, (1976) and involved the initial rotation of the measurement systems, followed by the translation toward the desired origin. The origin of the coordinate system {*O*} at the time t was denoted as $o\left(t\right)=1/2\left(P_{R}\left(t\right)+P_{L}\left(t\right)\right)$ , where $P_{R}\left(t\right)$ and $P_{L}\left(t\right)$ were the 3D position of the RASI and LASI joints at time t , respectively. Two unit vectors can be determined by $V_{1}=\left(P_{R}\left(t\right)-P_{L}\left(t\right)\right)/P_{R}\left(t\right)-P_{L}\left(t\right)$ and $V_{2}=\left(o\left(t\right)-o\left(t-1\right)\right)/o\left(t\right)-o\left(t-2\right)$ . Accordingly, $\left\{O\right\}=\left[x_{h},y_{h},z_{h}\right]$ can be denoted by

$$x_{h}=V_{1},$$ (2)

$$y_{h}=\frac{V_{1}\times V_{2}}{\left\|V_{1}\times V_{2}\right\|},$$ (3)

$$z_{h}=\frac{x_{h}\times y_{h}}{\left\|x_{h}\times y_{h}\right\|}.$$ (4)

Commonly, the 3D limb skeleton can be represented by three link segments: *Upper Trunk*, *Thigh*, and *Shank*. The 3D position vector between the right and left shoulder was denoted with  u . The 3D position of the right hip, knee, and ankle as joints was presented as  δ , α , and β , respectively. The upper trunk, right thigh, and shank as three links were represented as $\vec{U}=u-o$ , $\vec{T}=\alpha -\delta$ , and $\vec{S}=\alpha -\beta$ , respectively, as shown in Figure 4B. The knee angle was illustrated in the equation as follows:

$$RTS=arccos\frac{\vec{T}\cdot \vec{S}}{\left\|\vec{T}\right\|\times \left\|\vec{S}\right\|}.$$ (5)

Furthermore, we calculated the joint angles between each link segment $\left\{\vec{U},\vec{T},\vec{S}\right\}$ with respect to the normal vectors of the sagittal, coronal, and transverse planes $\left\{x_{h},y_{h},z_{h}\right\}$ and indicated as RUX , RTX ,  RSX ,  RUY , RTY ,  RSY ,  RUZ , RTZ , and  RSZ .

It should be noted that we estimated aforementioned features within each gait cycle, and the gait cycle was defined as the time interval between two consecutive heel-strike events. A heel-strike was the contact points between the heel and surface. We calculated gait events of right heel-strikes in marker motion capture and camera data by independently applying the different methods to each set of data. For marker data, we found one derived time series helpful for improving the detection of the gait cycle. The time series was the x-coordinates of the right ankles. Heel-strikes were defined by the time points of positive peaks in the anterior–posterior ankle trajectories. For the markerless-based system, heel-strike events were detected by visual inspection. The process was greatly aided by the identification of ankle key points obtained from the deep neural network model. In this letter, the RUX , RTX ,  RSX ,  RUY , RTY ,  RSY ,  RUZ , RTZ , and  RSZ were extracted at 2% increments throughout the entire cycle $\text{Φ}\left(t\right)\in {\mathbb{R}}^{K}\left(k=30\right)$ , for representing the gait pattern at each time stamp.

Moreover, we used sample entropy to measure the time series of joint angles in a gait cycle. Sample entropy is a nonlinear measurement way to analyze time series signals and is proposed by Richman and Moorman (2000). A higher sample entropy value indicates more randomness in time series, and lower value shows more self-similarity. The computational procedures of sample entropy (SampEn) are as follows (Pham, 2010):

Given a standardized (with zero mean and unit variance) time series {x(j);1≤j≤N} , *N* is the total number of data points.

Step 1 Construct subsequences of length m: $X_{m}\left(1\right),\;X_{m}\left(2\right),\cdots ,\;\;X_{m}\left(N-m\right)$ , where $X_{m}\left(i\right)=\left\{x\left(i+k\right);0\leq k\leq m-1\right\}$ and *m* is called as embedding dimension.

Step 2 Compute the distance between $X_{m}\left(i\right)$ and $X_{m}\left(j\right)$ , represented by $d\left(X_{m}\left(i\right),X_{m}\left(j\right)\right)$ , as:

$$d\left(X_{m}\left(i\right),\;X_{m}\left(j\right)\right)=max\left\{\left|x\left(i+k\right)-x\left(j+k\right)\right|;0\leq k\leq m-1,\;1\leq i,\;j\leq N-m,\;i\neq j\right\}$$ (6)

Step 3 Calculate the probability that any vector $X_{m}\left(j\right)$ which is similar to $X_{m}\left(i\right)$ within *T* as follows:

$$C_{i}\left(m,T\right)=\frac{n_{i}\left(m,T\right)}{N-m+1},i=1,\cdots ,\;N-m+1,$$ (7)

where ${\text{n}}_{i}\left(m,T\right)$ is the number of vectors $X_{m}\left(j\right)$ that are similar to $X_{m}\left(i\right)$ subject to the criterion of similarity: $d\left(X_{m}\left(i\right),\;X_{m}\left(j\right)\right)\leq T$ .

Step 4 Calculate

$$\emptyset \left(m,T\right)=\frac{1}{N-m+1}\sum_{i=1}^{N-m+1}C_{i}\left(m,T\right).$$ (8)

Step 5 Set m=m+1 and repeat steps 1–4.

Step 6 Calculate the sample entropy as

$$SampEn\left(N,m,T\right)=-ln\frac{\emptyset \left(m+1,T\right)}{\emptyset \left(m,T\right)}.$$ (9)

Hence, sample entropy is the negative natural logarithm of condition probability, without allowing self-matches. To calculate sample entropies of those time series, it is important to determine the appropriate values of the parameters m and T *.* Usually, the constant value of m is 1 or 2, T value ranging from 0.1 SD to 0.25 SD (SD is the standard deviation of time series) (Lake et al., 2002). In this experiment, we selected m=2 and T=0.2SD for each gait cycle data.

### Statistical analysis

Reliability and validity are central characteristics that define the quality of measurement methods and the test result potential for application in research and clinical practice (Michelini et al., 2020). First, we needed to assess the test–retest reliability within markerless and marker-based motion analysis systems, using intra-class correlation coefficients [ICC (C, 1)]. ICCs were determined as follows: almost perfect, 0.81–1.0; substantial, 0.61–0.80; moderate, 0.41–0.60; fair, 0.21–0.40; slight, 0.00–0.20 (Koo and Li, 2016).

Next, to assess the potential difference in joint angles among different measurement systems, an independent-sample t-test was used, when the variable conformed to the normality and homogeneity of variance simultaneously. If the variable did not conform to the normality or homogeneity assumption, the Wilcoxon nonparametric test was used for the difference analysis (Liang et al., 2021). In the event of a statistically significant main effect, we performed post-hoc pairwise comparisons with Bonferroni corrections.

Furthermore, we calculated standard errors of measurements (SEM) and intra-class correlation coefficients [ICC (A, 1)] with 95% confidence intervals (CI) of each joint angle to assess correlations and consistency. The smallest detectable change (SDC) was calculated as $1.96*\sqrt{2}*SEM$ . SDC can be regarded as the smallest change between any two steps that cannot be attributed to measurement errors (Furlan and Sterr, 2018). In addition, agreements between the joint angles at maximum flexion obtained from the markerless and marker-based systems were assessed by Bland–Altman analysis, which permits the delineation of systematic analysis (Bland and Altman, 1986). *p*-values less than 0.05 were deemed statistically significant. Data preprocessing, algorithms, and statistical analyses were implemented using Python (version 3.5).

## Results

### Participant characteristics

The data for gender and age did not conform to the normality (*p* < 0.05); therefore, the Wilcoxon nonparametric tests were used for difference analysis. The data of mass, height, and BMI were consistent with the normality and homogeneity assumptions (*p* > 0.05); therefore, an independent-sample t-test was used for the difference analysis. As shown in Table 1, the elderly group demonstrated a larger BMI than the young group (elderly: 24.17 ± 2.81, young: 21.53 ± 2.11, and *p* = 0.009). Moreover, age showed significant differences (elderly: 56.60 ± 2.53, young: 27.27 ± 4.31, and *p* = 0.000). It was indicated that strategies used for the control of gait deviation related to age between healthy young and elderly adult play an important role (Mehdizadeh, 2018).

**TABLE 1:** Comparison of characteristics between elderly and young groups.

	Elderly	Young	Normality	Homogeneity	Difference
Gender (men, %)	40%	67%	0.000*	0.478	0.217
Age (years)	56.60 ± 2.53	27.27 ± 4.31	0.000*	0.108	0.000*
Mass (kg)	61.28 ± 8.41	61.50 ± 8.59	0.163	0.662	0.947
Height (cm)	159.2 ± 8.41	168.73 ± 6.63	0.200	0.485	0.002*
BMI (kg/m^2^)	24.17 ± 2.81	21.53 ± 2.11	0.067	0.198	0.009*

Significant results are indicated with *.

### The reliability of motion analysis system

Mean values ±standard deviation and intra-class correlation coefficients for sample entropy of each joint angle test-retested using camera and Vicon are described in Table 2. There were moderate ICCs of the data obtained by marker motion capture (ICCs = 0.466–0.741 and *p* < 0.05). Similarly, ICCs of the data obtained by markerless motion capture were moderate (ICCs = 0.506–0.734 and *p* < 0.05). The reliability was confirmed on the joint angles measured using a single camera.

**TABLE 2:** Statistic property for marker and markerless motion analysis during test–retest.

	Markerless motion analysis		Marker motion analysis	
	Bias	ICC (95% CI; *p*-value)	Bias	ICC (95% CI; *p*-value)
*RTS*	0.032	0.734 [(0.592, 0.826); 0.000]	0.033	0.715 [(0.563, 0.814); 0.000]
*RUX*	0.015	0.714 [(0.561, 0.813); 0.000]	0.029	0.715 [(0.563, 0.814); 0.000]
*RTX*	0.075	0.617 ([0.405, 0.752]; 0.000)	0.078	0.611 [(0.391, 0.7500; 0.000]
*RSX*	0.044	0.649 [(0.380, 0.791); 0.000]	0.051	0.741 [(0.599, 0.833); 0.000]
*RUY*	−0.082	0.603 [(0.244, 0.775); 0.000]	−0.086	0.684 [(0.292, 0.837); 0.000]
*RTY*	0.066	0.610 [(0.394, 0.748); 0.000]	0.076	0.512 [(0.245, 0.684); 0.000]
*RSY*	0.104	0.519 [(0.253, 0.689); 0.000]	0.101	0.486 [(0.211, 0.665); 0.000]
*RUZ*	−0.040	0.658 [(0.476, 0.777); 0.000]	−0.40	0.699 [(0.537, 0.804); 0.000]
*RTZ*	0.169	0.506 [(0.148, 0.703); 0.000]	0.179	0.466 [(0.082, 0.678); 0.000]
*RSZ*	0.089	0.652 [(0.428, 0.784); 0.000]	0.095	0.581 [(0.317, 0.738); 0.000]

### The validity of motion analysis system

**FIGURE 5:**
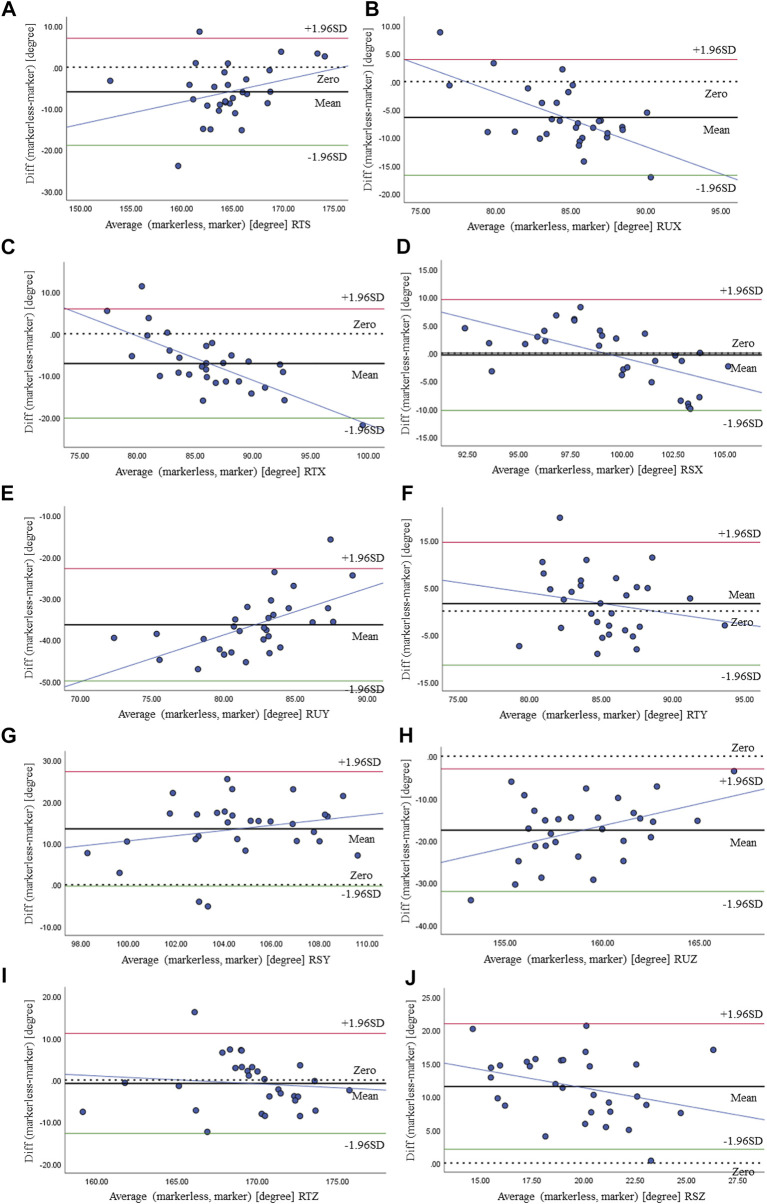
Bland–Altman plot of RTS **(A)**, RUX **(B)**, RTX **(C)**, RSX **(D)**, RUY **(E)**, RTY **(F)**, RSY **(G)**, RUZ **(H)**, RTZ **(I)**, and RSZ **(J)** at maximum flexion and comparison between the markerless- and marker-based systems.

All gait parameters were normally distributed and showed homogeneity of variance. Criterion validity, analyzed by the comparisons of joint angles results extracted from video recordings and results generated by the marker motion capture system, is shown in Table 3 and illustrated by the Bland–Altman plot (Figure 5).

**TABLE 3:** Comparison of characteristics between marker and markerless motion analysis.

	SDC	Bias (95% CI; *p*-value)	ICC (95% CI; *p*-value)
*RTS*	0.053	0.034 [(0.000, 0.068); 0.315]	0.726 [(0.579, 0.821); 0.000]
*RUX*	0.071	0.018 [(−0.019, 0.056); 0.325]	0.716 [(0.565, 0.815); 0.000]
*RTX*	0.055	0.081 [(0.036, 0.127); 0.399]	0.644 [(0.454, 0.768); 0.000]
*RSX*	0.064	0.047 [(0.008, 0.087); 0.174]	0.760 [(0.632, 0.844); 0.000]
*RUY*	0.054	−0.098 [(−0.123, −0.074); 0.000]	0.765 [(0.639, 0.847); 0.000]
*RTY*	0.040	0.084 [(0.045, 0.124); 0.000]	0.613 [(0.406, 0.748); 0.000]
*RSY*	0.044	0.108 [(0.054, 0.162); 0.000]	0.538 [(0.291, 0.699); 0.000]
*RUZ*	0.060	−0.046 [(−0.080, −0.013); 0.008]	0.694 [(0.530, 0.800); 0.000]
*RTZ*	0.058	0.185 [(0.130, 0.240); 0.000]	0.595 [(0.378, 0.736); 0.000]
*RSZ*	0.061	0.102 [(0.056, 0.145); 0.000]	0.665 [(0.485, 0.782); 0.000]

We calculated the sample entropy of joint angles across the stride cycle that was averaged for the walking bout of each individual participant, shown in Table 3. The SDC showed small values, with ranges between 0.040 and 0.071. It was indicated that small real differences between measurements could be detected by our method. Bias for sample entropy of joint angles between measurements was also shown to be over small, 0.034 for RTS, 0.018 for RUX, 0.081 for RTX, and 0.047 for RSX, and they were not statistically different (*p* > 0.05). This is supported by intra-class correlation coefficients that were substantial and the ICC estimates exceeded 0.61, indicating excellent reliability. Only reliability for RSY [95% CI = (0.291, 0.699)] and RTZ [95% CI = (0.378, 0.7360)] was lower, but still on a level of good-to-excellent reliability (*p* < 0.05). The Bland–Altman plots for each joint angle at maximum flexion obtained from the markerless and marker-based systems are provided in Figure 5. The *x*-axis and *y*-axis represent the average and difference between the outputs of the two methods, respectively. Thick and dotted lines denote the mean and zero of difference, respectively. The green and red lines represent the upper and lower limits of 95%, respectively, indicating most data are distributed within this range. The blue line shows that the difference between the two methods increases/decreases as the angle increases. The thick line was close to the dotted line on RTS, RUX, RTX, RSX, RTY, and RTZ, indicating higher consensus among the results of the two methods, otherwise on RUY, RSY, RUZ, and RSZ. This was consistent with the results given in Table 3.

## Discussion

In this study, the markerless pose estimation algorithm, OpenPose combined with 3DPoseNet, and signal processing techniques were used to evaluate a non-invasive method capable of capturing gait parameters. The main findings show that the proposed methods for extracting gait angles possess their own good validity and reliability. Several previous studies have used markerless-based analysis to study gait patterns of walking or other human movements (Michelini et al., 2020; Nakano et al., 2020; Ota et al., 2020). Our findings were consistent with these reports in that 3D markerless pose estimation in providing quantitative information about human movement is very promising.

First, ICC (C, 1) indicates the test–retest reliability within markerless- and marker-based systems was in almost complete agreement. At joint angles *RTS*, *RUX*, *RTX*, and *RSX*, there was no significant difference between the markerless- and marker-based motion analysis systems and the ICC (A, 1) was high enough. However, *RUY*, *RTY*, *RSY*, *RUZ*, *RTZ*, and *RSZ* were fair. One of the reasons is that there are differences in measurement methods of sample entropy of angles between markerless- and marker-based systems. While a marker-based system consisted of several cameras to form three-dimensional motion data, a markerless-based motion analysis system combined with deep algorithms to provide three-dimensional coordinates based on one camera. Therefore, the rotation motion has not been accurately measured. Another reason is that *RUY*, *RTY*, *RSY*, *RUZ*, *RTZ*, and *RSZ* were defined as the coronal and transverse plane angle of the normal vector relative to the upper trunk, right thigh, and shank. In the experiment, we acquired a dataset from stationary video camera recordings of healthy human gait, with sagittal plane views. It is possible that camera angles would likely affect results.

Some limitations of this study should be noted. First, some sources of error may be intrinsic to 3D markerless pose estimation. First and most obvious, it is difficult to track human movements frame-by-frame perfectly from the video. For example, the left and right segments could interchange or disappear in OpenPose. In this situation, our 3DPoseNet algorithm cannot predict 3D pose from a failed detector output. Second, the markerless-based identification system is unlikely to be equivalent to the marker landmarks. While marker placement depends on manual palpation of bony landmarks, a markerless-based system relies on visually labeled generalized key points. The placement of motion capture markers also owns some degree of error. These errors can affect the validity of the precision evaluation of a markerless-based system. Despite these limitations, the marker motion capture has been recognized as the ground truth (Nakano et al., 2020). As a result, the proposed method for evaluating a markerless-based system can be considered to be reasonable.

We did not pre-estimate the sample size. However, it exceeded what is commonly required for reliability studies (Koo and Li, 2016), since 30 subjects were instructed to walk on three times for the sake of collecting enough information to perform the analysis. Our approach also includes manual marking of the contact points between the heel and surface by visual inspection, which we register in order to accumulate a database to refine gait events. In the next work, it may be possible to obtain more accurate video-based analyses by training gait-specific networks from coronal and transverse views. It may be beneficial to train networks that are specific to each population, such as elderly people who experience accidental falls or abnormal gait. The markerless-based analysis described in the current study is promising for future applications. Such a method can classify different gait types and automatically extract quantitative gait information from the video.

## Conclusion

This study demonstrated the potential for combining OpenPose and 3DPoseNet markerless pose estimation algorithms to identify gait pathology. Given economic and time constraint problems, we have gained several insights from this exercise: 1) laboratory-based optical motion capture is a reasonable baseline predictor, while 3D markerless pose estimation networks were close to the ground_truth statistically significantly; 2) quantitative evaluations indicate that our proposed workflow trained on experimental movements can be generalized to non-experimental-specific poses; 3) correlation between the quantified results of network convergence support our initial hypothesis that learning a mapping from images to predict kinematics gait parameters is feasible; 4) the test–retest reliability within the device was in almost complete agreement. It was indicated that our video analysis could be used as a quantitative assessment of gait outside of a clinic. For predicting abnormal gait patterns or fall risk, future work should also include elderly people who experienced a fall.

## Data Availability

The raw data supporting the conclusion of this article will be made available by the authors, without undue reservation.
